# Protein Kinase A-induced tamoxifen resistance is mediated by anchoring protein AKAP13

**DOI:** 10.1186/s12885-015-1591-4

**Published:** 2015-08-14

**Authors:** Cristiane Bentin Toaldo, Xanthippi Alexi, Karin Beelen, Marleen Kok, Michael Hauptmann, Maurice Jansen, Els Berns, Jacques Neefjes, Sabine Linn, Rob Michalides, Wilbert Zwart

**Affiliations:** 1 Division of Molecular Pathology, the Netherlands Cancer Institute, Amsterdam, The Netherlands; 2 Division of Psychosocial Research and Epidemiology, the Netherlands Cancer Institute, Amsterdam, The Netherlands; 3 Department of Medical Oncology, Josephine Nefkens Institute and Cancer Genomics Center, Erasmus Medical Center Rotterdam, Rotterdam, The Netherlands; 4 Division of Cell Biology, the Netherlands Cancer Institute, Amsterdam, The Netherlands; 5 Department of Medical Oncology, the Netherlands Cancer Institute, Amsterdam, The Netherlands

## Abstract

**Background:**

Estrogen Receptor alpha (ERα)-positive breast cancer patients receive endocrine therapy, often in the form of tamoxifen. However, resistance to tamoxifen is frequently observed. A signalling cascade that leads to tamoxifen resistance is dictated by activation of the Protein Kinase A (PKA) pathway, which leads to phosphorylation of ERα on Serine 305 and receptor activation, following tamoxifen binding. Thus far, it remains elusive what protein complexes enable the PKA-ERα interaction resulting in ERα Serine 305 phosphorylation.

**Methods:**

We performed immunohistochemistry to detect ERαSerine 305 phosphorylation in a cohort of breast cancer patients who received tamoxifen treatment in the metastatic setting. From the same tumor specimens, Agilent 44 K gene expression analyses were performed and integrated with clinicopathological data and survival information. *In vitro* analyses were performed using MCF7 breast cancer cells, which included immunoprecipitations and Fluorescence Resonance Energy Transfer (FRET) analyses to illustrate ERα complex formation. siRNA mediated knockdown experiments were performed to assess effects on ERαSerine 305 phosphorylation status, ERα/PKA interactions and downstream responsive gene activity.

**Results:**

Stratifying breast tumors on ERα Serine 305 phosphorylation status resulted in the identification of a gene network centered upon AKAP13. AKAP13 mRNA expression levels correlate with poor outcome in patients who received tamoxifen treatment in the metastatic setting. In addition, AKAP13 mRNA levels correlate with ERαSerine 305 phosphorylation in breast tumor samples, suggesting a functional connection between these two events. In a luminal breast cancer cell line, AKAP13 was found to interact with ERα as well as with a regulatory subunit of PKA. Knocking down of AKAP13 prevented PKA-mediated Serine 305 phosphorylation of ERα and abrogated PKA-driven tamoxifen resistance, illustrating that AKAP13 is an essential protein in this process.

**Conclusions:**

We show that the PKA-anchoring protein AKAP13 is essential for the phosphorylation of ERαS305, which leads to tamoxifen resistance both in cell lines and tamoxifen-treated breast cancer patients.

**Electronic supplementary material:**

The online version of this article (doi:10.1186/s12885-015-1591-4) contains supplementary material, which is available to authorized users.

## Background

Breast cancer is the most frequently diagnosed malignancy in women. Since 75 % of all breast tumors express Estrogen Receptor alpha (ERα), tumor growth is considered to be dependent on the activity of this hormone-induced transcription factor. Thereby, treatment is focused on inhibiting the function of ERα. One of the most frequently prescribed drugs in endocrine treatment is tamoxifen. Tamoxifen competes with ERα’s natural ligand estradiol for binding to the ligand-binding pocket of the receptor. Tamoxifen forces the receptor in an alternative conformation as compared to estradiol, thereby preventing recruitment of coregulators to the complex, which are essential for ERα-driven transcription [1]. Although tamoxifen is considered a highly successful drug, resistance to treatment is common. Resistance to tamoxifen treatment can occur through a multitude of mechanisms, including activation of the MAP kinase pathway [2–4] or overexpression of PAK1 [5], SRC1 [6], SRC3 [7] and ErbB2 [8].

An alternative mechanism of tamoxifen resistance is mediated by activation of the Protein Kinase A (PKA) pathway [9]. Decreased expression of a regulatory component of the PKA complex, PKA-RIα, was found to correlate with a non-favorable prognosis in breast cancer patients treated with tamoxifen [10]. We could confirm these data [9], and found *in vitro* that PKA-RIα knockdown enhances breast cancer cell proliferation in the presence of tamoxifen. In addition, we showed in the same study that the major site PKA-responsive phosphorylation site on ERα is a serine residue found at position 305. This phosphorylation leads to a conformational arrest within the receptor and results in an agonistic response of the otherwise inhibitory compound tamoxifen. In this altered conformation ERα re-orientates its C-terminus towards its coactivators, which prevents a dissociation of RNA Polymerase II from the complex that is normally observed in tamoxifen treated cells, thereby resulting in transcriptional activation in the presence of tamoxifen [11]. PKA-stimulated MCF-7 breast cancer cells express a unique repertoire of genes that are differentially expressed as compared to tamoxifen or PKA-activating cAMP treatment alone. [12] This potentially underlies a cell biological response for this pathway to tamoxifen resistance. In accordance, we recently reported S305 phosphorylated ERα to bind a unique set of promoters regulating transcriptional programs involved in tamoxifen resistance [13].

In addition to *in vitro* data, in breast cancer patients treated with tamoxifen for metastatic disease, the phosphorylation status of ERαS305 was found to be indicative for poor outcome [14]. Moreover, ERαS305 phosphorylation was found to be a predictive marker for tamoxifen resistance in pre- [14, 15] and postmenopausal patients [16]. However, it remains elusive what regulates the ERα/PKA interaction, which is an essential step in the ERαS305 phosphorylation pathway.

PKA is a multi-protein complex. The inactivated PKA complex is composed of a catalytic subunit (PKAcat) that associates, in an inactive state, to its regulatory subunits (PKA-RI and RII). After the regulatory subunits bind cAMP, the catalytic subunit can dissociate from the complex to phosphorylate its substrates. There are many known PKA targets, and in order to achieve substrate-specificity, PKA activity needs to be locally confined. Localized PKA activation can occur in multiple ways. Intracellularly, cAMP levels are unevenly and dynamically distributed, [17, 18] which can partly be explained by the tethering of phosphodiesterases (PDEs) at distinct subcellular domains [19–21]. Alternatively, PKA activity can be locally confined by A-kinase anchoring proteins (AKAPs). The regulatory subunits of PKA can interact with AKAP family members, which determine the subcellular localization of the PKA complex. Next to associating with PKA subunits, AKAPs can also physically interact with the PKA substrates. This way, activated PKA can act locally and directly on its substrates, thereby orchestrating substrate specificity. AKAPs possess a PKA-anchoring domain composed of a 14–18 residue long amphipathic helix [22], by which they interact with a hydrophobic groove formed by 4 alpha-helical structures on the PKA-RII subunit [23–26]. At least 50 AKAPs have been identified, with varying expression levels among different tissues and with their own unique intracellular localizations [27]. A number of these AKAPs have been reported to correlate with the occurrence of different cancer subtypes, including AKAP3 (ovarian cancer) [28, 29], AKAP4 (multiple myeloma) [30], AKAP9 (breast cancer) [31], AKAP10 (breast cancer) [32] and AKAP13 (colorectal cancer [33] and breast cancer [34]). Thus far, it remains unknown which AKAP family member is responsible for enabling the PKA-induced phosphorylation of Serine 305 on ERα, resulting in tamoxifen resistance.

Here, we studied the enrichment of PKA-associated molecular pathways in a cohort of breast cancer patients, which received tamoxifen in a metastatic setting. Stratifying tumors on ERαS305 phosphorylation status resulted in the identification of a molecular pathway involving AKAP13, suggesting a functional link between AKAP13 levels and PKA-induced ERα phosphorylation. AKAP13 expression levels correlated with a poor outcome after tamoxifen treatment in breast cancer patients and correlated with ERαS305 phosphorylation status. *In vitro* experiments could illustrate that AKAP13 interacts with ERα as well as the regulatory subunit of PKA, and that AKAP13 expression is essential for PKA-induced ERαS305 phosphorylation. In summary, we demonstrate here that the PKA-mediated phosphorylation of ERα at Serine 305, which leads to tamoxifen resistance, requires the PKA-anchoring protein AKAP13.

## Methods

### Tissue culture, plasmids, antibodies, shRNA and siRNA

MCF-7 cells were cultured in DMEM medium, supplemented with 8 % fetal-bovine serum and standard antibiotics. The following antibodies were used: AKAP13 (Bethyl A301-404A-1), ERα (Santa Cruz sc-543), ERαS305P (Millipore, clone 124.9.4), PKA RII (Abcam ab57414), PKA catalytic-α (Cell Signalling 4782), beta-actin (Abcam, ab8229) and AKAP95 (Bethyl, A301-061A). 3 days prior to cell biological analyses, cells were switched to phenol-red free DMEM, supplemented with 5 % charcoal-treated serum. For siRNA targeting of AKAP13 and AKAP95, a smartpool of four unique siRNAs was applied (Thermo Scientific Dharmacon) using the manufacturers’ protocols. Cells were subsequently cultured for 72 h on hormone-deprived phenol-red free DMEM. The expression vector for ERα-CFP was previously described [9]. The expression vectors encoding for the regulatory subunit and the YFP-tagged catalytic subunit of PKA were a kind gift from dr. Kees Jalink (NKI). shRNAs were provided by the NKI robotics and screening core facility.

### Cell proliferation assays

MCF-7 cells were switched into phenol-red free DMEM supplemented with 5 % charcoal-treated serum. After 24 h, cells were transfected with siAKAP13, siAKAP95 or siCntrl. After 2 days the cells were seeded in 48 well plates at a density of 10^4^ cells/ well in phenol-red free DMEM, supplemented with 5 % charcoal-treated serum. Compounds were introduced the following day and cells were allowed to grow in the presence of the compounds for 2 weeks. The compounds tested were 17β-estradiol (10^−8^ M), 4-OH-tamoxifen (10^−7^ M) and Fulvestrant (10^−7^ M), while incubation in the presence of vehicle alone (DMSO) was used as a control. After 2 weeks cells were fixed with methanol and stained by a 0.2 % crystal violet solution. Finally, the dye was solubilized in a weak acetic acid solution and the optical density measured at 590 nm using the Infinite® 200 reader (TECAN). Alternatively, growth was assessed by MTT using the manufacturers recomendations (Sima-Aldrich).

### Immunohistochemistry

For immunohistochemistry, tissue microarrays (TMAs) were used from formalin fixed-paraffin-embedded tumor tissue. Antigen retrieval was performed using citrate buffer citrate buffer (10 mM, pH 6.0). Citrate buffer was pre-heated and slides were subsequently added for 15 min (microwave 300 W). The ERαS305P antibody staining was performed overnight in 1:20 dilution (Millipore # 124-9-4). No lower cut-off for positivity was applied, and any ERαS305 positive signal in the entire slide was scored as positive. This mode of scoring was consistent as previously described for this antibody [14, 35].

### Immunoprecipitaion

MCF-7 cells were grown in DMEM until ~40 % confluency was achieved. Cells were subsequently hormone depleted by culturing in heat inactivated charcoal-treated serum-containing medium for 3–4 days to block all ERα activity. Prior to immunoprecipitation, cells were washed and lysed according to standard protocols. Immunoprecipitation was performed overnight with anti PKA RII, AKAP13, ERα or negative control anti FLAG-M2 (Sigma). Thereafter, the beads were washed and prepared for Western blot analysis.

### Quantitative RT-PCR

For quantitative RT-PCR analyses, cells were seeded in 12 well plates and hormone-deprived for 3 days. After six hours of hormonal treatment, RNA was isolated using Trizol (Life Technologies) using the manufacturers protocols. cDNA was generated using SuperScript III (Life Technologies) using the manufacturers protocols. Primers used for QPCR were for XBP1 (GGGAAGGGCATTTGAAGAAC (FWD); ATGGATTCTGGCGGTATTGA (REV)) and TFF1 (ATCGACGTCCCTCCAGAAGA (FWD); TGGGACTAATCACCGTGCTG (REV)). As housekeeping gene, GAPDH (GCCATCAATGACCCCTTCAT (FWD); TGACAAGCTTCCCGTTCTCA (REV) was used.

### FRET imaging

FRET was performed using Fluorescence Lifetime Imaging Microscopy (FLIM) as we applied before [36]. Cells were seeded on coverslips and mounted in bicarbonate-buffered saline. Imaging was performed in a heated tissue culture chamber at 37C and under 5 % CO_2_. FLIM experiments were performed using a LEICA DM-IRE2 microscope equipped with a Lambert Instruments frequency domain lifetime attachment. CFP was excited at 430 nm. Emission was collected at 450–490 nm using a CCD camera. FLIM measurements were performed using U2Os cells, transfected with ERα-CFP, PKA-cat-YFP and a non-tagged PKA-regulatory subunit. Cells were co-transfected with siAKAP13 or siControl. Calculated CFP lifetimes were referenced to Rhodamine-G6 which was set at 4.11 nsec, and internally calibrated using cocultured CFP containing MelJuso cells for which the lifetime was set to 2.7 nsec, as we applied before [36]. Donor FRET efficiency (E_D_) was calculated as E_D_ = 1- (lifetime cell of interest/ lifetime reference cell). Pairwise analysis was performed for each cell before and after treatment with 10 μM forskolin for 1 h, where E_D_ under CTS conditions was set to 1 for each experiment.

### Patient series

The patient series used in this paper has been previously described in detail [15]. This study was performed in accordance with the Code of Conduct of the Federation of Medical Scientific Societies in the Netherlands (http://www.fmwv.nl) and has been approved by the local medical ethics committee of the Netherlands Cancer Institute. The use of anonymous or coded left over material for scientific purposes is part of the standard treatment agreement with patients and therefore informed consent was not required according to Dutch law [37].

For the analysis of pathway enrichment in ERαS305P positive breast cancer patients we used a series of breast cancer patients who were treated with tamoxifen for metastatic disease as was previously described [14, 35]. In brief, this cohort from The Netherlands Cancer Institute (NKI) consists of a consecutive series of 158 breast cancer patients, who were selected according to the following criteria: 1) invasive ERα-positive breast carcinoma, 2) no adjuvant systemic treatment, 3) development of relapse before 2002, for which first-line tamoxifen mono-therapy was given. Tamoxifen was administered according to the national guidelines of that time. Of this series gene expression data and IHC data on ERαS305P were available for 58 tumors. For the analyses of AKAP13 levels in patients, we used the same NKI series. Agilent 44 K array gene expression data was available for 66 patients. In addition, we used gene-expression data available for a second cohort, from the Erasmus Medical Centre (EMC) [38]. This EMC cohort consists of 112 patients with primary operable, invasive, ERα–positive breast cancer, diagnosed between 1981 and 1992, who developed disease recurrence and were treated with tamoxifen as first-line treatment. For the EMC series, Agilent 44 K array gene expression data were available for 40 patients. For all gene expression analyses, data from both cohorts were combined. For IHC analyses, only the NKI samples were used.

### Gene expression analysis

The analysis for pathway enrichment in ERαS305P positive patients and Agilent 44K data was previously described [35]. Data analyses were performed using BRB array tools (version 3.6). First, using the gene set expression comparison tool, 302 pathways (as defined by Biocarta) were analyzed. The evaluation of pathways that are differentially expressed between ERαS305P positive and ERαS305P negative samples was done using a functional class scoring analysis as previously described [35]. Fisher’s Least Square (LS) summary statistic (10,000 permutations) was used to test which pathways were differentially expressed in ERαS305P-positive tumors. First, a *P*-value was computed for each gene in a pathway. Then the set of *P*-values for a pathway was summarized by the LS summary statistics. For a set of N genes, the LS summary statistic (LS = ∑^N^_i=1_ (−log(p_i_))/N) was defined as the mean negative natural logarithm of the P-values of the appropriate single gene univariate test. Second, we related the pathways to PKA using the Cancer Genome Anatomy Project (http://cgap.nci.nih.gov/Genes/GeneFinder) and 27 pathways out of the 302 were found to be related to PKA. Next, we tested whether the list of significant pathways as defined by the LS statistic (*P* < 0.05) was enriched for PKA-related pathways using Fisher’s Exact test.

### Statistics

Time to tumour progression (TTP) was considered the primary endpoint measured from the start of tamoxifen administration and until treatment was ended because of tumour progression. TTP was estimated according to the Kaplan–Meier method for four different AKAP13 probes, segmenting the continuous variable in two groups (low and high) with an equal number of events. In addition we tested the four different AKAP13 probes for trend, using the significance of the coefficient for the continuous AKAP13 variable. For the probe with the most significant test for trend, we compared TTP in the NKI cohort for two subgroups (low and high) by uni- and multi-variate Cox proportional hazards regression. Clinico-pathological characteristics (only available for NKI cohort) according to AKAP13 levels were compared using Fisher’s exact, Mann-Witney *U* test and the Chi square test for trend. Data were analyzed using SPSS 15.0.

## Results

### AKAP pathway enrichment in ERαS305-P positive breast cancer patients

**Fig. 1:**
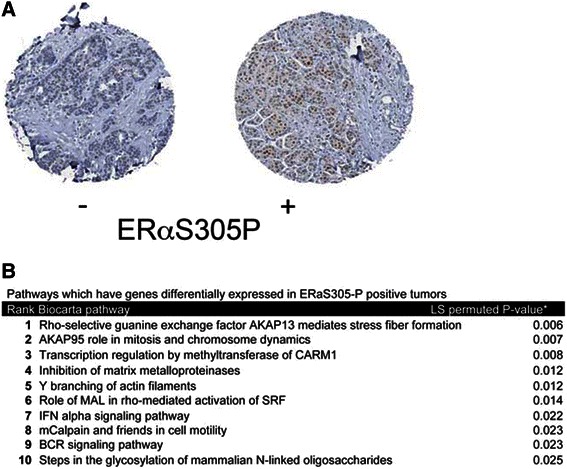
Molecular pathway enrichment in ERαS305P positive tumors. **a** Tissue microarrays, stained for ERαS305P were analysed and subgrouped into staining negative (left panel) or positive (right panel) for ERαS305P signal. Expression array data from these subgroups were clustered and pathway enrichement analyses were performed on the expression array data from each subgroup. **b** AKAP13 and AKAP95 were found to be the most significantly enriched pathways

Serine 305 phosphorylation on ERα was found to be a predictive marker for tamoxifen resistance in breast cancer patients [14, 15]. To define which cell biological factors may be causally involved in the PKA-induced phosphorylation of ERαS305, we performed pathway enrichment analyses from a cohort of breast cancer patients, which received tamoxifen for metastatic disease [14, 15]. The samples were stained for ERαS305P and scored for positivity by immunohistochemistry (Fig. 1a). Available expression array data [15] was reanalyzed using Biocarta pathway analysis. Among the 19 pathways differentially and significantly enriched in these two patient groups, five were found to be PKA-related. The top two differentially regulated pathways involved AKAP13 and AKAP95 signaling cascades. Since both of these proteins are members of the PKA-anchoring protein family, PKA-substrate specificity regulated by AKAP levels may be causally linked with ERαS305 phosphorylation status, and thus tamoxifen resistance.

### AKAP13 interacts with ERα and PKA-RII and is essential for PKA-mediated S305 Phosphorylation of ERα

**Fig. 2:**
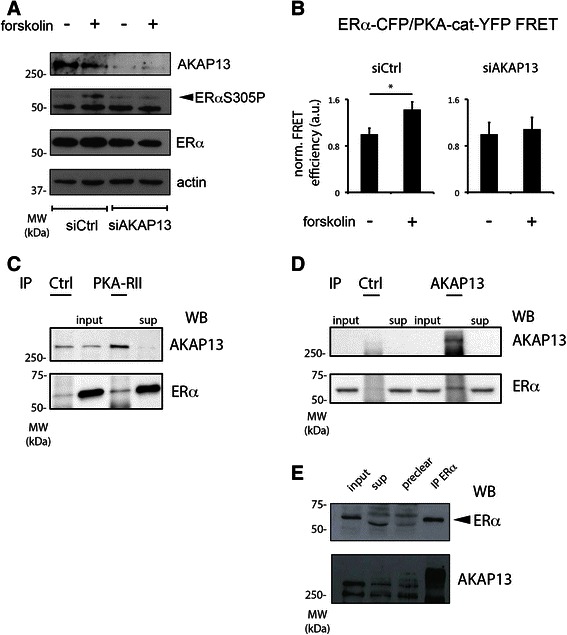
AKAP13 is required for PKA-induced ERαS305 phosphorylation and interacts with ERα and PKA-RII. **a** siRNA targeting AKAP13 prevents PKA-induced ERαS305 phosphorylation. MCF-7 breast cancer cells were transfected with an siRNA targeting AKAP13 or a control siRNA, after which the cells were treated for 1 h with 10 μM forskolin or left untreated. Samples were analysed by SDS-PAGE and Westernblotting, probing with antibodies detecting AKAP13, ERαS305P, ERα or actin as a loading control. **b** AKAP13 is required for a forskolin-enhanced ERα/PKA-cat interaction. Estrogen Receptor-negative U2OS cells were transfected with CFP-tagged ERα, YFP-tagged PKA catalytic subunit and a non-tagged PKA regulatory RII subunit. In addition, cells were transfected with an siRNA targeting AKAP13 or a control siRNA. Energy transfer from the CFP to the YFP fluorophore was measured in the same cell before and after 1 h of 10 μM forskolin treatment, and the average value prior to treatment was set on 1. N > 10. Bars indicate SEM. A student’s *T*-test was performed; *p* < 0.05. **c, d, e** AKAP13, ERα and PKA-RII form a complex. MCF-7 cells were hormone deprived for 3 days to deplete activated ERα transcriptional processes. Following that, cells were lysed for immunoprecipitations, directed at PKA-RII (**c**), AKAP13 (**d**), ERα (**e**) or a negative control protein. In addition, input and supernatant (sup) samples were taken. Western blots were probed with antibodies detecting AKAP13, ERα and PKA-RII

Since AKAP13 and AKAP95 signaling cascades were significantly enriched in the ERαS305P positive patient subgroup, we tested *in vitro* whether this enrichment also implies a causal role for either of these two proteins in PKA-mediated ERα Serine 305 phosphorylation. siRNA-mediated knockdown of AKAP13 and AKAP95 was performed in MCF-7 breast cancer cells (Fig. 2a and Additional file 1: Figure S1, respectively). Cells were either treated or not treated with the PKA-activating compound forskolin for 1 h prior to cell lysis. As expected and previously observed [39], PKA activation by forskolin treatment sufficed to induce phosphorylation of ERα on Serine 305. Knocking down AKAP13 prevented the forskolin-induced increase in ERαS305P signal, implying that AKAP13 is required for the phosphorylation of ERα at this site. This effect was not observed after knock down AKAP95, and Serine 305 on ERα could still be phosphorylated upon PKA activation (Additional file 1: Figure S1). Furthermore, both AKAP13 as well as AKAP95 were not essential for MCF-7 cell proliferation arrest after tamoxifen treatment while proliferation under E2 conditions was slightly increased (Additional file 2: Figure S2). These data suggest that AKAP13 is not a crucial player in normal ERα biology and tamoxifen-response in absence of PKA activation.

AKAP13 is known to be an anchoring protein of PKA, binding both PKA-RII as well as PKA-substrates [27]. Thus, in order for AKAP13 to enable ERαS305 phosphorylation, it needs to interact with ERα as well as members of the PKA complex. To illustrate such a complex, we used two parallel but distinct approaches; FRET (Fig. 2b) and co-immunoprecipitation (Fig. 2c, d, e). FRET, or Fluorescence Resonance Energy Transfer, is the radiation-free energy transfer from a donor fluorophore to a suitable acceptor, and is highly dependent on the distance between them [40]. This technology enables the determination of protein-protein interactions in living cells in real time, and we previously applied this approach to monitor interactions between ERα and its coregulator SRC-1 [11]. To prevent dilution of any potential FRET signal by endogenous proteins, the experiments were performed in ERα-negative U2OS cells. Cells were transfected with ERα-CFP, the catalytic subunit of PKA (PKA-cat)-YFP, and a non-tagged regulatory subunit of PKA to prevent a constitutive activation of PKA-cat. In addition, cells were transfected with an siRNA targeting AKAP13 or a control siRNA. When the cells were treated with the PKA-activating compound forskolin, an increase in FRET signal was observed, implying an increased interaction between ERα and PKA-cat. This increase was not observed when AKAP13 was knocked down, suggesting that the interaction was lost.

In addition to the biophysical analyses, co-immunoprecipitations were performed. To perform these experiments analogous to the FRET assays, and to block all ERα activity that may affect protein complex formation, cells were hormone-deprived for 3 days prior to onset of the experiment. PKA-RII was immunoprecipitated and its association with ERα and AKAP13 was assessed (Fig. 2c). Both these factors were enriched in the PKA-RII IP fraction as compared to IgG control. Note that the AKAP13 protein levels were decreased in the supernatant fraction after IP. The PKA-RII regulatory subunit overlapped with the IgG heavy chain, preventing us to reliably identify this protein as a part of the complex. We also performed an immunoprecipitation for AKAP13 and again identified ERα as part of the complex (Fig. 2d). The reciprocal experiment was performed as well, and AKAP13 could be identified after immunoprecipitating ERα (Fig. 2e). These data collectively indicate that the interaction of ERα with the PKA complex is mediated by AKAP13 and AKAP13 is essential for the PKA-mediated ERαS305 phosphorylation.

### AKAP13 expression correlates with a non-favorable outcome after tamoxifen treatment and with ERαS305P positivity in breast cancer patients

**Fig. 3:**
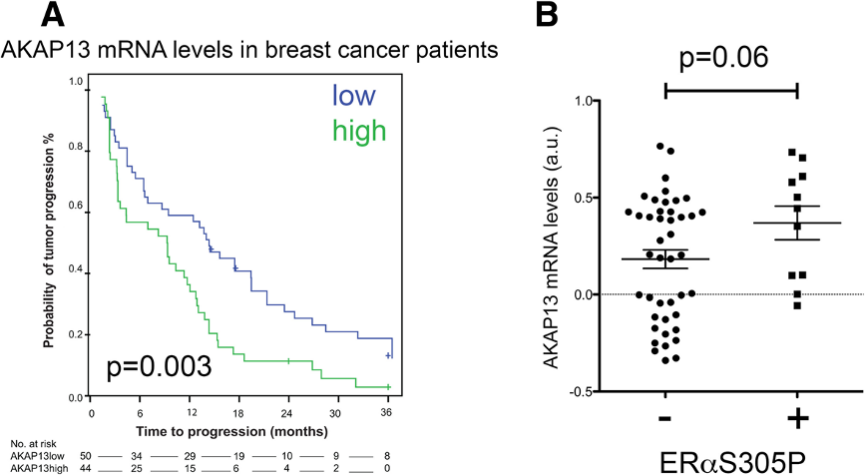
AKAP13 mRNA levels, survival and ERαS305P. **a** AKAP13 expression levels were analyzed and correlated with progression-free survival of a cohort of breast cancer patients treated for metastatic disease. **b** Tumors were stratified on the basis of ERαS305P positivity, the AKAP13 mRNA levels of each sample were analyzed. A Student’s *T*-test was performed; *p* = 0.06

Our findings indicated that expression of AKAP13 in tissue culture experiments is essential for the PKA-induced Serine 305 phosphorylation. Next, we investigated whether AKAP13 levels by themselves correlate with an unfavorable response to treatment in breast cancer patients. Therefore, tumor samples were analyzed, in which patients with metastatic disease were treated with tamoxifen. Time to tumor progression (TTP) was estimated according to the Kaplan–Meier method for AKAP13 expression, segmenting the continuous variable in two groups (low and high) with equal numbers of patients (Fig. 3a). Four different AKAP13 probes were available from the Agilent 44 K array data, and identical analyses were performed for all the remaining probes (Additional file 3: Figure S3). Of these four probes, 3 had significant log rank tests. In addition, we tested the four different AKAP13 probes for trend, using the significance of the coefficient for the continuous AKAP13 variable (Additional file 4: Table S1) and found a significant test for trend for probe 1 (*p* = 0.022). We then compared clinico-pathological characteristics for two subgroups (low and high) of this probe, Table 1. AKAP13 high tumours were more often lymph node positive (*p* = 0.033) and there was a trend towards negative progesterone receptor status (*p* = 0.071). Using a univariate Cox proportional hazard model we found a hazard ratio of 2.10 for patients with high AKAP13 levels (*p* = 0.007). Other clinico-pathological characteristics that were significant in the univariate analysis were ERα level (HR 0.20, *p* = 0.0002) and PR status (HR 0.45 *p* = 0.004) (Additional file 4: Table S2). Combining these variables in the multivariate analysis resulted in a hazard ratio for AKAP13 of 1.84 (*p* = 0.031) (Additional file 4: Table S3).

**Table 1:** Patient characteristics distributed by AKAP13 status

Variable	AKAP 13 low		AKAP 13 high		*P*-value
	n	%	n	%	
**Total**	38		28		
**Median age at surgery**					
Years (range)	64 (38–85)		61(37–82)		0.211^£^
**WHO type carcinoma**					
IDC	33	86.8	23	82.1	0.246*
ILC	4	10.5	4	14.3	
IDC + DCIS	1	2.6	0	0.0	
Unknown	0	0.0	1	3.6	
**Tumor diameter**					
<= 20 mm	20	52.6	14	50.0	1.000
>20 mm	18	47.4	14	50.0	
**Lymph node status**					
Negative	29	76.3	14	50.0	0.033
Positive	8	21.1	13	46.4	
Unknown	1	2.6	1	3.6	
**Histological grade** ^**a**^					
Grade I	9	23.7	12	42.9	0.374*
Grade II	19	50.0	7	25.0	
Grade III	10	26.3	8	28.6	
Unknown	0	0.0	1	3.6	
**Estrogen receptor**					
Low (≥10 % < 75 %)	7	18.4	10	35.7	0.160
High (≥75 %)	30	78.9	18	64.3	
Unknown	1	2.6	0	0.0	
**Progesterone receptor**					
Negative	11	28.9	15	53.6	0.074
Positive	26	68.4	13	46.4	
Unknown	1	2.6	0	0.0	
**HER2 status**					
Negative	30	78.9	27	96.4	0.366
Positive	4	10.5	1	3.6	
Unknown	4	10.5	0	0.0	
**ER305phosporylation**					
Negative	25	78.1	18	66.7	0.510
Positive	5	15.6	6	22.2	
Unknown	2	6.3	3	11.1	

*P* values: patients with unknown values were omitted. *P* values were calculated using the Fisher’s exact test, except for *Chi square test for trend and £ Mann–Whitney *U* test

*IDC* invasive ductal carcinoma, *ILC* invasive lobular carcinoma, *DCIS* ductal carcinoma *in situ*

^a^According to Bloom and Richardson

If AKAP13 is indeed required for the PKA-mediated Serine 305 phosphorylation in breast cancer patients, the expression levels of AKAP13 would be expected to correlate with ERαS305P positivity. Unfortunately, using multiple antibodies against AKAP13, the IHC signal was high in most cells with a minimal dynamic window, precluding a reliable categorization of the samples based on IHC. Therefore, we resorted to analyzing AKAP13 mRNA expression derived from expression arrays that were generated from the samples of the metastatic cohort, and compared those with the ERαS305P signal as detected by IHC performed on the same tumors (Fig. 3b). The slides were scored on the basis of ERα S305P positivity, after which the mRNA levels of AKAP13 were analyzed. High AKAP13 mRNA levels were found enriched in the ERα S305P positive subgroup with a p value of 0.06. These data illustrate that AKAP13 mRNA levels correlate with a poor outcome after tamoxifen treatment as well as ERαS305 phosphorylation status in breast cancer patients. For AKAP95, which did not affect ERαS305 phosphorylation *in vitro* (Additional file 1: Figure S1), no correlation between ERαS305P IHC and AKAP95 mRNA levels was found (Additional file 5: Figure S4).

### AKAP13 knockdown decreases ERα-responsive gene expression in PKA-driven tamoxifen-resistant cells

**Fig. 4:**
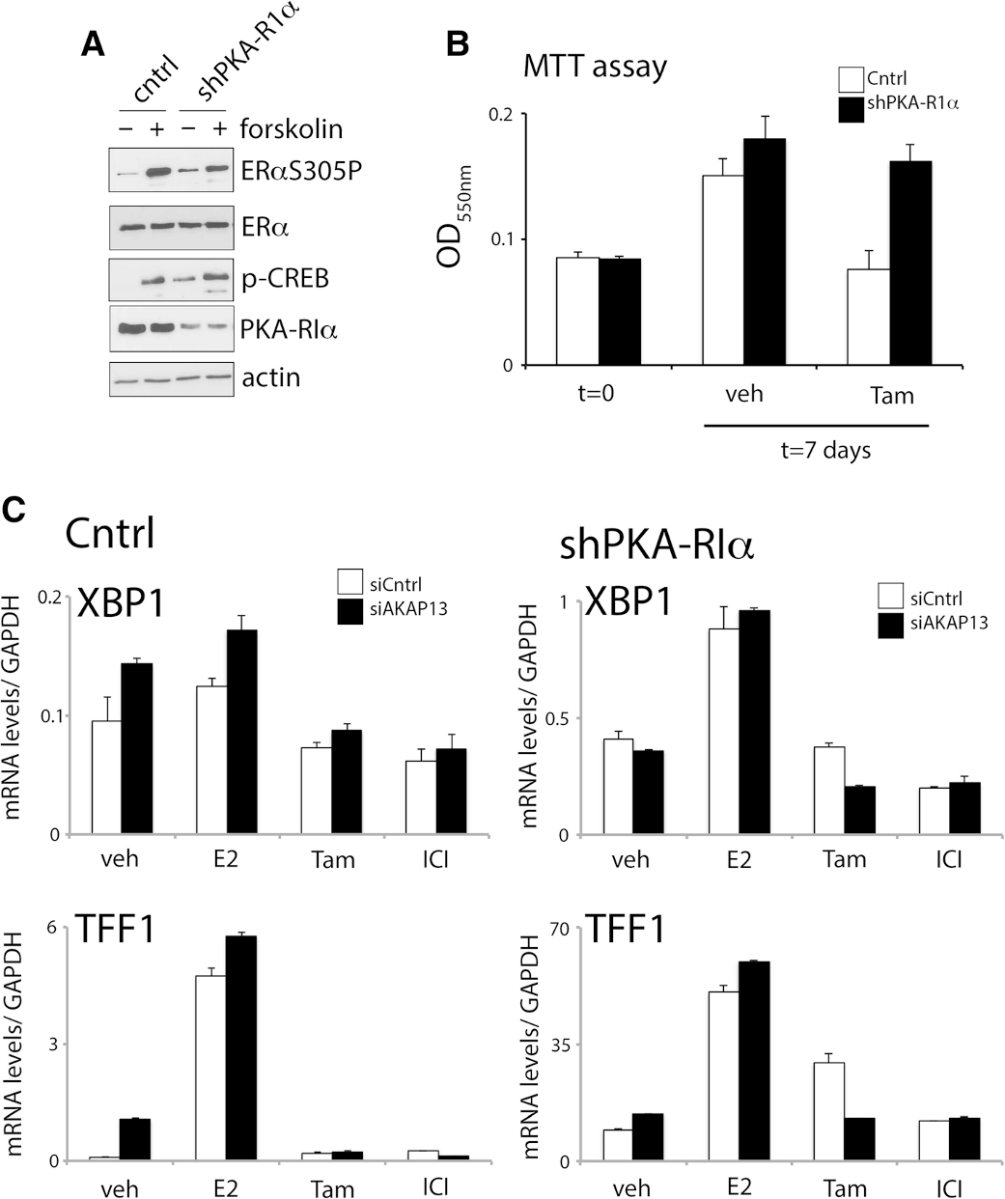
AKAP13 is required for tamoxifen-driven gene expression in PKA-activated cells. **a** Activation of the PKA pathway by shRNA-PKA-RIα. Western blot analysis of ERαS305P, ERα, phospho-CREB and PKA-RIα. Actin was used as loading control. **b** PKA-RIα knockdown gives rise to tamoxifen resistance. Control (white) or PKA-RIα knockdown (black) cells were used. An MTT assay was performed after cells were seeded in hormone-deprived medium (t = 0) and cultured for a week in the absence or presence of tamoxifen. Error bars indicate SD values from six independent measurements. **c** RT-QPCR analyses for Control (left) or shPKA-RIα (right) cells. Cells were hormone-deprived and transfected with siCntrl (white) or siAKAP13 (black). Three days after transfection, cells were incubated with estradiol (E2), tamoxifen (Tam), Fulvestrant (ICI) or DMSO control (veh). Expression levels of XBP-1 (top) and TFF1 (bottom) were analysed. Error bars indicate SD values from 3 independent measurements

AKAP13 interacts with ERα and is required for ERαS305 phosphorylation. Does AKAP13 knockdown also result in a decrease of ERα-driven gene expression in tamoxifen-resistant cells? For this, we generated an MCF-7 derivative cell line that is tamoxifen-resistant through enhanced PKA activity. An shRNA was used targeting the regulatory subunit of PKA, PKA-RIα, as was performed before [9]. Knocking down PKA-RIα activated the PKA pathway, as shown by a phosphorylation of CREB, and increased ERαS305P levels (Fig. 4a). Furthermore, while control cells could be effectively blocked in their cell proliferation using tamoxifen, this was not the case when PKA-RIα was knocked down (Fig. 4b). Now that we have established a PKA-driven tamoxifen resistant cell line, the next question was whether the agonistic features of tamoxifen could be blocked by targeting AKAP13. Since PKA activation stimulates cell proliferation both in an ERα-dependent as well as an ERα-independent fashion [9], interpretation of cell proliferation in shPKA-RIα cells following siAKAP13 may be challenging. To focus the analysis on ERα-functioning in the context of PKA-driven tamoxifen resistance, we therefore decided to perform RT-QPCR for two well-annotated ERα-responsive genes: TFF1 and XBP1 (Fig. 4c). For both these genes, shPKA-RIα knockdown greatly increased ERα action. Importantly, siAKAP13 did not affect TFF1 and XBP1 expression in control tamoxifen-treated cells, where expression levels were comparable to those found for the full ERα antagonist Fulvestrant (ICI). In cells with PKA-RIα knockdown, XBP1 and TFF1 levels were considerably higher under tamoxifen conditions as compared to Fulvestrant conditions. Targeting AKAP13 however decreased TFF1 and XBP1 levels in tamoxifen-treated shPKA-RIα cells, now comparable to those levels found under Fulvestrant conditions. These data indicate that activation of PKA increases ERα-driven gene expression under tamoxifen conditions, which can be reverted by knocking down AKAP13.

Combining the clinical information and cell biological analyses confirms that AKAP13 is part of the same complex as ERα and PKA and describes how AKAP13 expression is essential for PKA-induced ERαS305 phosphorylation, leading to tamoxifen resistance.

## Discussion

Resistance to endocrine treatment is a significant clinical challenge. Endocrine treatment is exclusively prescribed to patients with luminal breast tumors that comprise approximately 70 % of all breast cancer cases. Luminal tumors are typically positive for Estrogen Receptor alpha (ERα), and are considered to grow dependent on the activity of this transcription factor. About 50 % of breast cancer cases without distant metastatic disease can be cured by surgery alone [14] and endocrine treatment would not be essential for this patient subpopulation to prevent a relapse. The remaining group of patients does require additional treatment to prevent a relapse and endocrine treatment achieves this goal in 50 % of these patients. The remaining group is considered to be resistant to endocrine treatment and would therefore require alternative drug treatment in order to prevent patient relapse. Importantly, tumors that do relapse on one type of endocrine treatment, may still respond to alternative endocrine agents [41]. This implies that cross-resistance is not an intrinsic feature of anti-estrogen resistance and matching the right patient with the right drug is key to further improving breast cancer patient treatment outcome.

Kinases play a central role in endocrine resistance, with many (receptor tyrosine) kinases being differentially expressed or differentially activated in endocrine treatment resistant breast cancer. Examples of these include EGFR [42], ErbB2 [8], IGF-1R [43], PKA [9] and PAK1 [5, 15]. The current study illustrates that not only global kinase activity, but also locally confined kinase action, which leads to substrate specificity, can play a role in endocrine resistance. PKA is considered a highly promiscuous kinase with the capacity of phosphorylating 64 substrates identified thus far [44]. To achieve substrate specificity, PKA localization (via its specific interactions with AKAPs [27]) and activation (through local cAMP concentrations [17, 18]) are tightly controlled. In the studied cohort of breast cancer patients, we found AKAP95 as well as AKAP13 signaling pathways significantly enriched in the ERαS305P positive subpopulation. While AKAP95 knockdown did not influence PKA-induced ERαS305P levels *in vitro*, siRNA targeting AKAP13 did prevent ERαS305 phosphorylation. These data highlight the necessity for *in vitro* verification of findings based on correlations in patient subpopulations before drawing conclusions that suggest any causality.

**Fig. 5:**
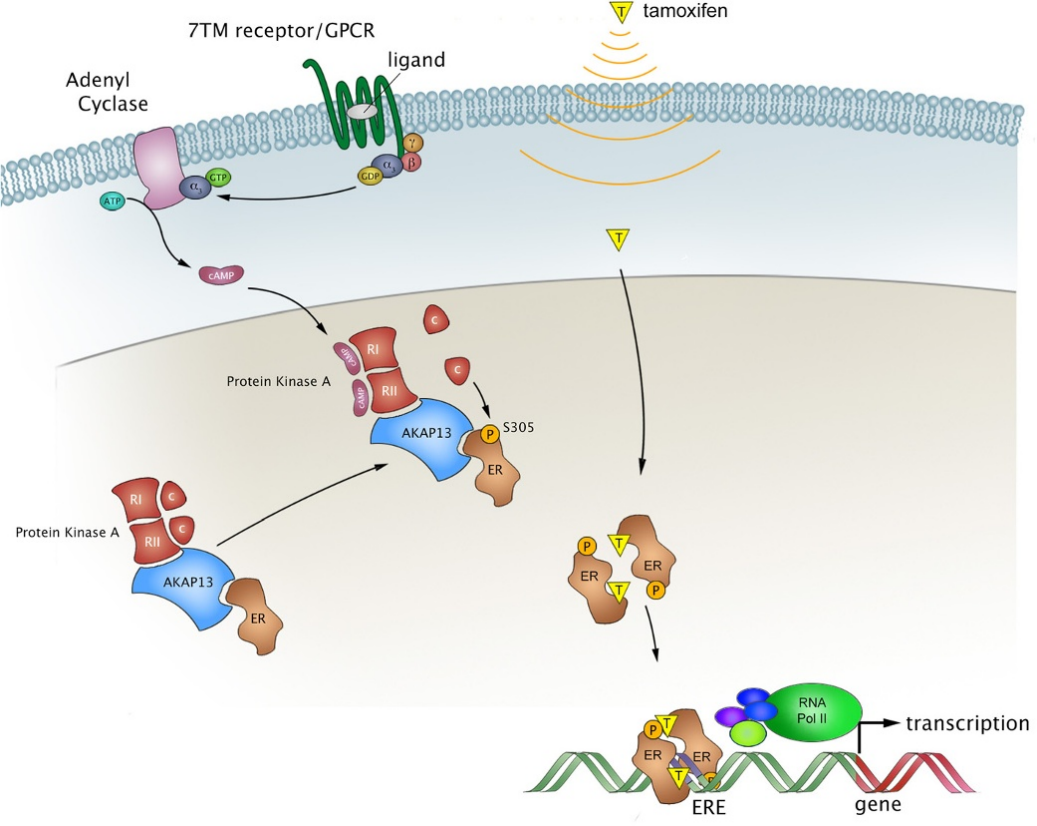
Model for PKA-mediated ERαS305 phosphorylation and the role of AKAP13 in this process. AKAP13 interacts with ERα as well as PKA-RII. This way, AKAP13 functions as a scaffolding protein, bringing together the PKA complex and its substrate, ERα. When members of the seven-transmembrane domain (7TM) receptors/ G protein-coupled receptors (GPCRs) are activated, this results in an activation of Adenyl Cyclase to generate cAMP. PKA is consequently activated, by the association of the PKA-regulatory subunits with cAMP. Subsequently, the catalytic subunit can dissociate from the complex and directly phosphorylate ERα at Serine 305. This phosphorylation at ERαS305P results in the recognition of tamoxifen as an ERα agonist, initiation of tamoxifen-driven gene transcription and consequently tamoxifen resistance

As a model for PKA-mediated ERαS305P phosphorylation, leading to tamoxifen resistance, we propose the following order of events (as illustrated in Fig. 5): AKAP13 interacts with ERα as well with PKA-RII. PKA-RII forms a complex with PKA-RI and the catalytic subunit of PKA, so that the entire PKA complex is indirectly associated to ERα. This interaction is present in the absence of PKA activation. When cAMP levels increase, cAMP can bind PKA-RI and PKA-RII, so that PKA-cat is dissociated from the complex. By virtue of the preformed protein complex that is mediated by AKAP13, the activated PKA-cat can locally act and phosphorylate ERαS305. When the phosphorylated ERα binds tamoxifen, ERα is selectively targeted to a distinct set of gene promoters to activate these genes, resulting in tamoxifen resistance [13].

Previous studies support a role of AKAP13 in ERα biology and PKA-mediated tamoxifen resistance. A polymorphism in AKAP13 (Lys526Gln) has been described to correlate with high-risk familial breast cancer [32, 34]. In addition, AKAP13 has been described to interact with ERα, enhancing its transcriptional potency [45]. Interestingly, AKAP13 has also been reported to integrate differential signalling cascades in addition to PKA, including 14-3-3, Rho, PKC and PKD [27]. AKAP13 brings PKC and PKD within the same complex, so that PKC can activate PKD [46]. In addition, PKA activity phosphorylates AKAP13, so that 14-3-3 can bind to the complex [47, 48]. This 14-3-3 association diminishes the Rho-GEF activity of AKAP13, lowering Rho activity [48]. Since PKA [15, 49], 14-3-3, PKC [50, 51] and now AKAP13 (this study) have all been implicated in tamoxifen resistance, this macromolecular complex could provide a central function for AKAP13 in tamoxifen non-responsiveness, which could potentially be exploited for pharmacological intervention.

Kinases are promising drug targets in cancer treatment. Still, when subcellular localization and activity are key determining factors rather than global activation status of a kinase, the pharmacological inhibition of the total kinase pool could potentially result in a significantly high level of toxicity. Inhibiting the specific interactions of kinases with their localization-confining factors may provide a highly specific inhibition of a kinase subset, while the total pool of such a kinase remains unaffected. With respect to the current study, the interface between ERα and AKAP13 could be a suitable drugable interaction, through which this form of tamoxifen resistance could potentially be inhibited.

## Conclusions

Breast cancer stratification on ERαS305 phosphorylation status led to the identification of AKAP13 as a potential mediator for this phosphorylation event on ERα. AKAP13 expression levels correlate with response to tamoxifen treatment in the metastatic setting. Tumors with high AKAP13 mRNA levels are enriched for ERαS305P positivity on IHC. Using cell lines, we have shown that AKAP13 interacts both with ERα and PKA-RII, which enables PKA-induced ERα phosphorylation and tamoxifen-driven gene expression in a tamoxifen-resistant cell line with elevated PKA activity. With this, AKAP13 plays a key function in enabling PKA-induced ERα phosphorylation and is causally involved in ERαS305P-mediated tamoxifen resistance.

## Additional files

Additional file 1: Figure S1. siRNA targeting AKAP95 does not influence PKA-induced ERαS305 phosphorylation. MCF-7 breast cancer cells were transfected with an siRNA targeting AKAP95 or a control siRNA, after which the cells were treated for 1 h with 10 μM forskolin or left untreated. Samples were analysed by SDS-PAGE and Western blotting, probing with antibodies detecting AKAP95 or actin as a loading control. (JPEG 96 kb)
Additional file 2: Figure S2. Knockdown of AKAP13 and AKAP95 does not impair MCF-7 cell proliferation in vehicle, E2, tamoxifen and Fulvestrant (ICI) treated cells. Cells were seeded in 48 well format, and transfected with siCntrl, siAKAP13 or siAKAP95. After 1 week, cells were processed for crystal violet cell quantifications. (PDF 219 kb)
Additional file 3: Figure S3. AKAP13 expression correlations with progression-free survival from all 4 available probes. (PDF 920 kb)
Additional file 4: Table S1. Univariate analysis for different AKAP13 probes. **Table S2.** Univariate analysis. **Table S3.** Multivariate analysis. (PDF 64 kb)
Additional file 5: Figure S4. AKAP95 expression does not correlate with progression-free survival (A) and 305P status (B). (PDF 409 kb)

## Abbreviations

AKAP: A-kinase anchoring protein
cAMP: Cyclic adenosine monophosphate
CREB: cAMP response element-binding protein
EGFR: Epidermal growth factor receptor
ERα: Estrogen Receptor alpha
ERaS305: Estrogen Receptor alpha Serine 305
ErbB2: avian erythroblastosis oncogene B 2
FLIM: Fluorescence lifetime imaging microscopy
FRET: Fluorescence resonance energy transfer
HR: Hazard rate
IGF-1R: Insulin-like growth factor 1 receptor
IHC: Immunohistochemistry
MCF-7: Michigan Cancer Foundation-7
PAK1: P21 protein (Cdc42/Rac)-activated kinase 1
PDEs: Phosphodiesterases
PKA: Protein Kinase A
PKA-RIα: Protein Kinase A regulatory subunit type I, alpha
PKA-RII: Protein Kinase A regulatory subunit type II
PKC: Protein kinase C
PKD: Protein kinase D
RT-PCR: Reverse transcription polymerase chain reaction
shRNA: short hairpin RNA
siRNA: small interfering RNA
SRC1: Steroid receptor coactivator 1
SRC3: Steroid receptor coactivator 3
TFF1: Trefoil factor 1
TTP: Time to tumour progression
XBP1: X-box binding protein 1
