# Supplementary figures and images for: Protein Kinase A-induced tamoxifen resistance is mediated by anchoring protein AKAP13
Source: BMC Cancer. 2015 Aug 14;15:588. doi: 10.1186/s12885-015-1591-4 (PMC4536754; doi:10.1186/s12885-015-1591-4)

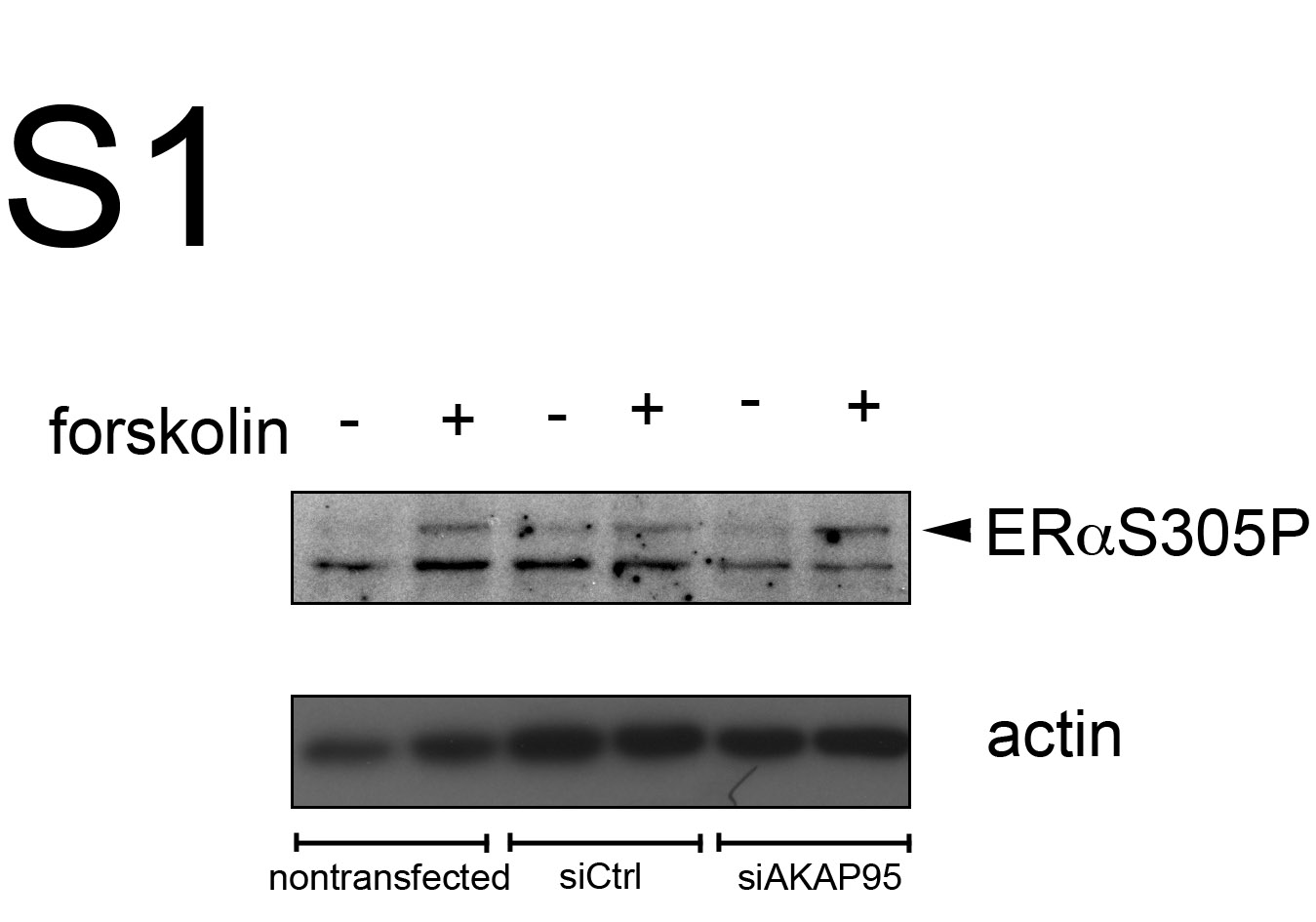

Supplement: Additional file 1: Figure S1. — siRNA targeting AKAP95 does not influence PKA-induced ERαS305 phosphorylation. MCF-7 breast cancer cells were transfected with an siRNA targeting AKAP95 or a control siRNA, after which the cells were treated for 1 h with 10 μM forskolin or left untreated. Samples were analysed by SDS-PAGE and Western blotting, probing with antibodies detecting AKAP95 or actin as a loading control. (JPEG 96 kb) [file 12885_2015_1591_MOESM1_ESM.jpeg]

# S2

## MCF7 cell proliferation

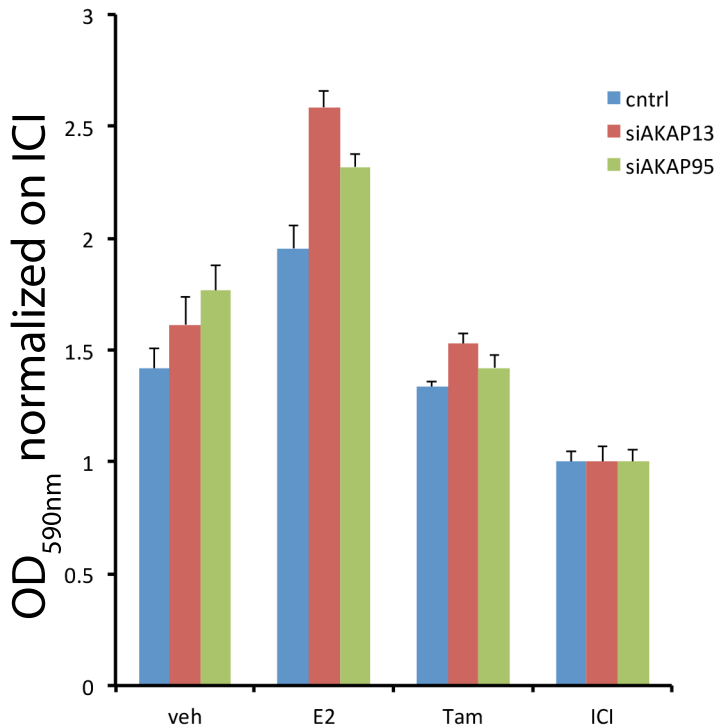

Supplement: Additional file 2: Figure S2. — Knockdown of AKAP13 and AKAP95 does not impair MCF-7 cell proliferation in vehicle, E2, tamoxifen and Fulvestrant (ICI) treated cells. Cells were seeded in 48 well format, and transfected with siCntrl, siAKAP13 or siAKAP95. After 1 week, cells were processed for crystal violet cell quantifications. (PDF 219 kb) [file 12885_2015_1591_MOESM2_ESM.pdf]

probe1

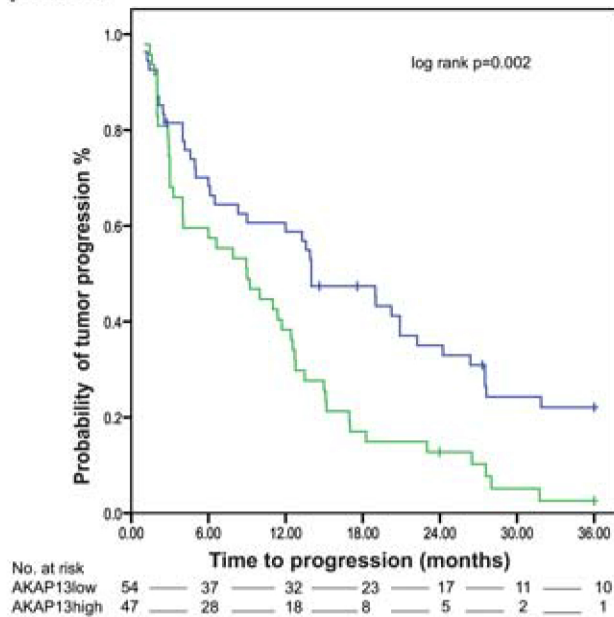

probe 2

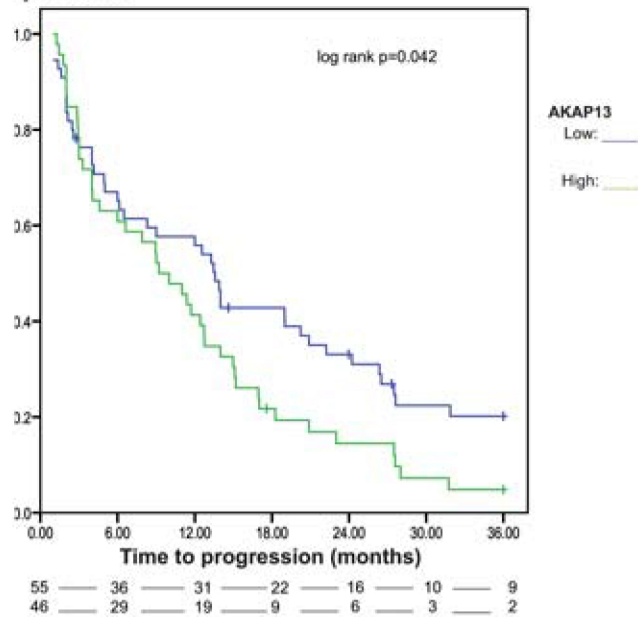

probe3

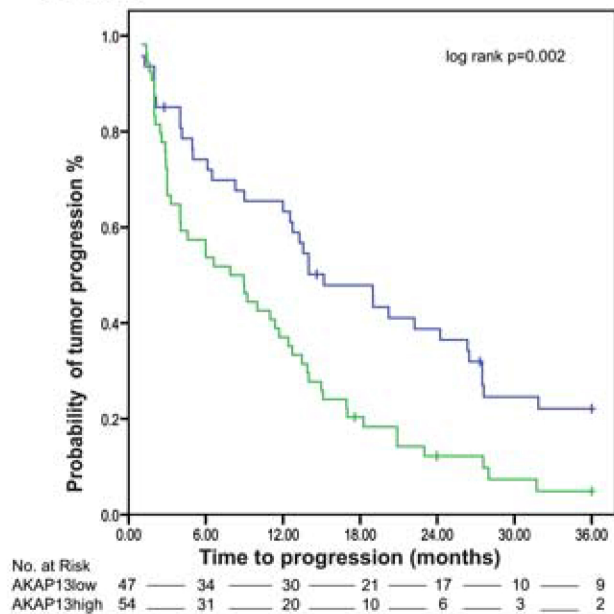

probe4

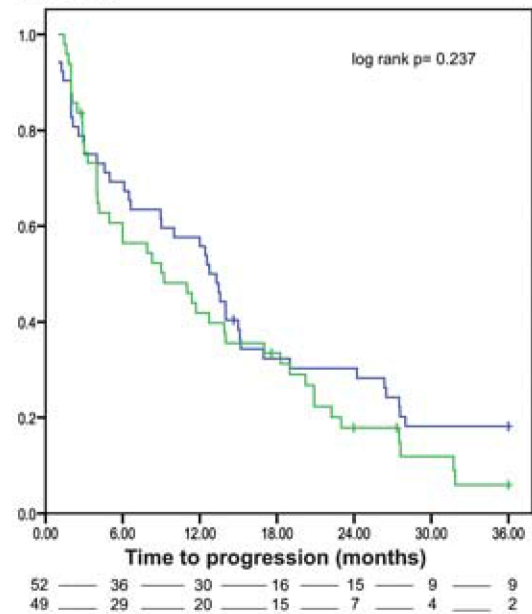

Supplement: Additional file 3: Figure S3. — AKAP13 expression correlations with progression-free survival from all 4 available probes. (PDF 920 kb) [file 12885_2015_1591_MOESM3_ESM.pdf]

# S4A

## AKAP95

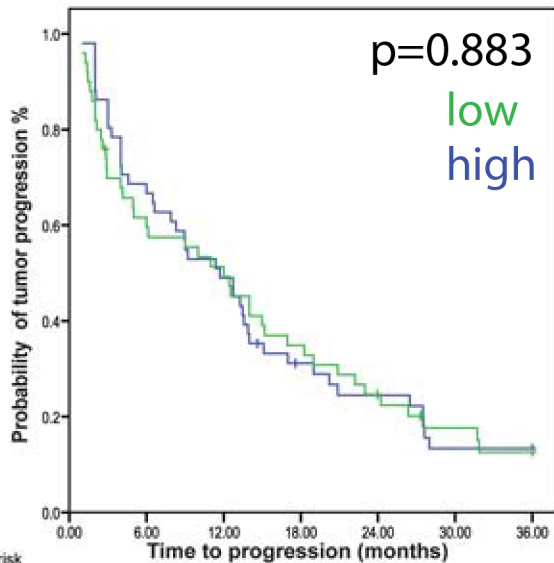

# B

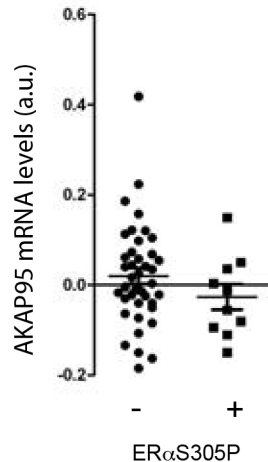

Supplement: Additional file 5: Figure S4. — AKAP95 expression does not correlate with progression-free survival (A) and 305P status (B). (PDF 409 kb) [file 12885_2015_1591_MOESM5_ESM.pdf]
