# Supplementary material for: Protein Kinase A-induced tamoxifen resistance is mediated by anchoring protein AKAP13
Source: BMC Cancer. 2015 Aug 14;15:588. doi: 10.1186/s12885-015-1591-4 (PMC4536754; doi:10.1186/s12885-015-1591-4)
Supplement: Additional file 4: Table S1. — Univariate analysis for different AKAP13 probes. Table S2. Univariate analysis. Table S3. Multivariate analysis. (PDF 64 kb) [file 12885_2015_1591_MOESM4_ESM.pdf]

Supplement table 1 Univariate analysis for different AKAP`probes

Variable	HR	95% CI	P-value
AKAP13Probe1 (A_24_P155761)	2.74	1.20-6.27	0.017
AKAP13Probe2 (A_24_P164337)	3.21	0.76-13.61	0.114
AKAP13Probe3 (A_23_P137276)	3.14	0.61-16.20	0.171
AKAP13Probe4 (A_24_P142095)	2.34	0.54-10.17	0.255
AKAP95 (A_23_P142380)	4.60	0.56-37.73	0.155

Univariate Cox regression analysis for risk of progression after tamoxifen treatment for metastatic disease. NKI and EMC patients are included. Continuous variable for 4 different AKAP13 probes are used.

Supplemental table 2. Univariate analysis

Variable	HR	95% CI	P-value
Estrogen receptor* (high vs low)	0.42	0.23-0.75	0.003
Progesterone receptor (positive vs negative)	0.46	0.27-0.78	0.004
AKAP13 (high vs low)	2.10	1.22-3.59	0.007
Tumor diameter (> 20 mm vs <= 20 mm)	1.39	0.83-2.33	0.216
Lymph node status (positive vs negative)	1.26	0.72-2.21	0.415
Histological grade (grade III vs grade I-II)	1.12	0.62-2.00	0.703
HER2 status (positive vs negative)	1.148	0.41-3.22	0.793

Univariate Cox regression analysis for risk of progression after tamoxifen treatment for metastatic disease. * Estrogen receptor low ≥ 10% < 75%), high (≥ 75%). Only NKI patients were analysed

Supplemental table 3. Multivariate analysis

Variable	HR	95% CI	P-value
Estrogen receptor* (high vs low)	0.36	0.19-0.66	0.001
Progesterone receptor (positive vs negative)	0.42	0.24-0.76	0.004
AKAP13 (high vs low)	1.79	1.02-3.15	0.044

Multivariate Cox regression analysis for risk of progression after tamoxifen treatment for metastatic disease. * Estrogen receptor low ≥ 10% < 75%), high (≥ 75%). Only NKI patients were analyzed.
